# Germline *BRCA1* mutation reprograms breast epithelial cell metabolism towards mitochondrial-dependent biosynthesis: evidence for metformin-based “starvation” strategies in *BRCA1* carriers

**DOI:** 10.18632/oncotarget.9732

**Published:** 2016-05-31

**Authors:** Elisabet Cuyàs, Salvador Fernández-Arroyo, Tomás Alarcón, Ruth Lupu, Jorge Joven, Javier A. Menendez

**Affiliations:** ^1^ ProCURE (Program Against Cancer Therapeutic Resistance), Metabolism and Cancer Group, Catalan Institute of Oncology, Girona, Catalonia, Spain; ^2^ Molecular Oncology Group, Girona Biomedical Research Institute (IDIBGI), Girona, Catalonia, Spain; ^3^ Unitat de Recerca Biomèdica, Hospital Universitari de Sant Joan, IISPV, Universitat Rovira i Virgili, Campus of International Excellence Southern Catalonia, Reus, Spain; ^4^ Institució Catalana d'Estudis i Recerca Avançats (ICREA), Barcelona, Spain; ^5^ Computational and Mathematical Biology Research Group, Centre de Recerca Matemàtic (CRM), Barcelona, Spain; ^6^ Departament de Matemàtiques, Universitat Autònoma de Barcelona, Barcelona, Spain; ^7^ Barcelona Graduate School of Mathematics (BGSMath), Barcelona, Spain; ^8^ Mayo Clinic, Department of Laboratory Medicine and Pathology, Division of Experimental Pathology, Rochester, MN, USA; ^9^ Mayo Clinic Cancer Center, Rochester, MN, USA

**Keywords:** BRCA1, hereditary breast cancer, breast cancer susceptibility, bioenergetics, glutamine

## Abstract

We hypothesized that women inheriting one germline mutation of the *BRCA1* gene (“one-hit”) undergo cell-type-specific metabolic reprogramming that supports the high biosynthetic requirements of breast epithelial cells to progress to a fully malignant phenotype. Targeted metabolomic analysis was performed in isogenic pairs of nontumorigenic human breast epithelial cells in which the knock-in of *185delAG* mutation in a single *BRCA1* allele leads to genomic instability. Mutant *BRCA1* one-hit epithelial cells displayed constitutively enhanced activation of biosynthetic nodes within mitochondria. This metabolic rewiring involved the increased incorporation of glutamine- and glucose-dependent carbon into tricarboxylic acid (TCA) cycle metabolite pools to ultimately generate elevated levels of acetyl-CoA and malonyl-CoA, the major building blocks for lipid biosynthesis. The significant increase of branched-chain amino acids (BCAAs) including the anabolic trigger leucine, which can not only promote protein translation *via* mTOR but also feed into the TCA cycle *via* succinyl-CoA, further underscored the anabolic reprogramming of *BRCA1* haploinsufficient cells. The anti-diabetic biguanide metformin “reversed” the metabolomic signature and anabolic phenotype of BRCA1 one-hit cells by shutting down mitochondria-driven generation of precursors for lipogenic pathways and reducing the BCAA pool for protein synthesis and TCA fueling. Metformin-induced restriction of mitochondrial biosynthetic capacity was sufficient to impair the tumor-initiating capacity of *BRCA1* one-hit cells in mammosphere assays. Metabolic rewiring of the breast epithelium towards increased anabolism might constitute an unanticipated and inherited form of metabolic reprogramming linked to increased risk of oncogenesis in women bearing pathogenic germline *BRCA1* mutations. The ability of metformin to constrain the production of mitochondrial-dependent biosynthetic intermediates might open a new avenue for “starvation” chemopreventive strategies in *BRCA1* carriers.

## INTRODUCTION

Germline mutations of the *BRCA1* gene confer a breast cancer risk in women 10- to 20-fold higher than in those with the wild-type gene [1–3]. Although hereditary tumors in women that carry *BRCA1* mutations account for only a small percentage (5–10%) of breast cancers [4], the risk of developing the disease throughout the lifetime is much higher (up to 85%) in *BRCA1* mutation carriers than in noncarriers. According to the “two-hit” hypothesis proposed more than 40 years ago by Knudson [5], individuals carrying a germline mutation in one copy of the *BRCA1* gene require just one additional mutation in the same gene in an otherwise normal breast epithelial cell for malignant transformation. However, *BRCA1*-mediated tumorigenesis apparently contradicts the original “two-hit” theory conformed by other familial cancer syndromes, in which consecutive deletion of two alleles accelerates tumorigenesis. Paradoxically, the absence of both *BRCA1* alleles in adult human cells induces cell proliferation defects that lead in the main to cell death. Moreover, the bi-allelic inactivation of *BRCA1* commonly observed in tumors of *BRCA1* cancer patients results in early embryonic lethality when reproduced in animal models [6–8]. This raises the question, how can tumor cells survive with loss of both *BRCA1* alleles?

Following biallelic, homozygous inactivation of *BRCA1*, pre-cancerous epithelial cells must amass additional genetic alterations on other genome guardian genes to survive. Accumulating evidence suggests that breast cancer predisposition upon inactivation of a single *BRCA1* allele is caused by the so-called phenomenon of haploinsufficiency associated with heterozygosity [9–20], which results in genomic instability in breast epithelial cells [13, 14, 17, 20]. This in turn may promote additional genetic changes in *BRCA1* heterozygous cells, including the acquisition of new mutations that will precede and be permissive with the loss of *BRCA1* (e.g., *p53*, *ATM* and *CHK2*). The requirement of this “extra-hit”, although incongruous from the viewpoint of familial tumorigenesis mediated by a tumor suppressor gene such as *BRCA1*, appears to enable cancer-prone *BRCA1* “one-hit” cells to evade the cell death processes that would otherwise occur upon loss of the remaining wild-type allele.

While studies to identify genetic alterations, particularly activating changes, are warranted to better understand how the properties of *BRCA1* haploinsufficiency influence the restricted tissue distribution of *BRCA1* tumorigenesis, it is important to consider that breast malignancy can occur early in women with a germline *BRCA1* mutation, whereas other *BRCA1* mutation carriers develop disease much later or not at all [21]. From a strictly genetic perspective, if genetic instability caused by loss of *BRCA1* allows the acquisition of mutations in critical checkpoint genes during puberty, this phenomenon would enable rare *BRCA1* null cells to escape death and proliferate, leading to early breast cancer onset. If a majority or all cells with somatic inactivation of the remaining wild-type *BRAC1* allele succumb to checkpoint-mediated cell death, tumors would occur much later in the life of a woman with an inherited *BRCA1* mutation. Alternatively, the incomplete penetrance associated with inherited *BRCA1* mutations might reflect the fact that non-genetic modifiers have an important role in determining cancer risk among *BRCA1* carriers. Although reproductive, dietary and lifestyle factors remain controversial with regards to their ability to influence *BRCA1*-related cancer risk [22–25], the common assumption that the fundamental mechanism(s) underlying breast cancer predisposition are expected to be different in *BRCA1* mutation carriers than in the general population further complicates the scenario.

By considering metabolic networks that could reconcile both genetic and nongenetic causal mechanisms in *BRCA1*-driven tumorigenesis [16, 26–30], we herein tested the challenging hypothesis that *BRCA1* haploinsufficiency drives metabolic rewiring in breast epithelial cells, acting as an early but suppressible “hit” that pushes *BRCA1* one-hit cells toward malignant transformation. On the one hand, metabolic analyses of human cancers are beginning to indicate that mitochondrial damage and altered metabolism can precede malignancy [31–33]. On the other hand, induction of genomic instability comes at the cost of significant stress, which obliges cells to modify their energy use to provide adaptation against genetic changes as well as to promote their survival and growth [34–36]. Thus, normal breast epithelial cells bearing a single inherited “hit” in *BRCA1* might become pre-equipped with a metabolic phenotype capable of supporting the high energetic and anabolic requirements for progression to a fully malignant phenotype. We present strong evidence for an unforeseen one-hit inherited metabolic trait linked to increased risk for oncogenesis in women with pathogenic germline *BRCA1* mutations that might be suppressible by the anti-diabetic drug metformin.

## RESULTS

### Mutation of a single *BRCA1* allele dramatically alters the metabolomic signature of normal-like breast epithelial cells

We utilized our recently developed targeted metabolomics platform coupling gas chromatography with quadrupole time-of-flight mass spectrometry and an electron impact source (GC-EI-QTOF-MS), which allows the simultaneous measurement of 30 selected metabolites representative of the catabolic and anabolic status of key metabolic nodes. These metabolites include not only representatives of glycolysis and the mitochondrial tricarboxylic acid (TCA) cycle, but also other biosynthetic routes such as pentose phosphate pathway, amino acid metabolism and *de novo* fatty acid biogenesis [37, 38].

**Figure 1:**
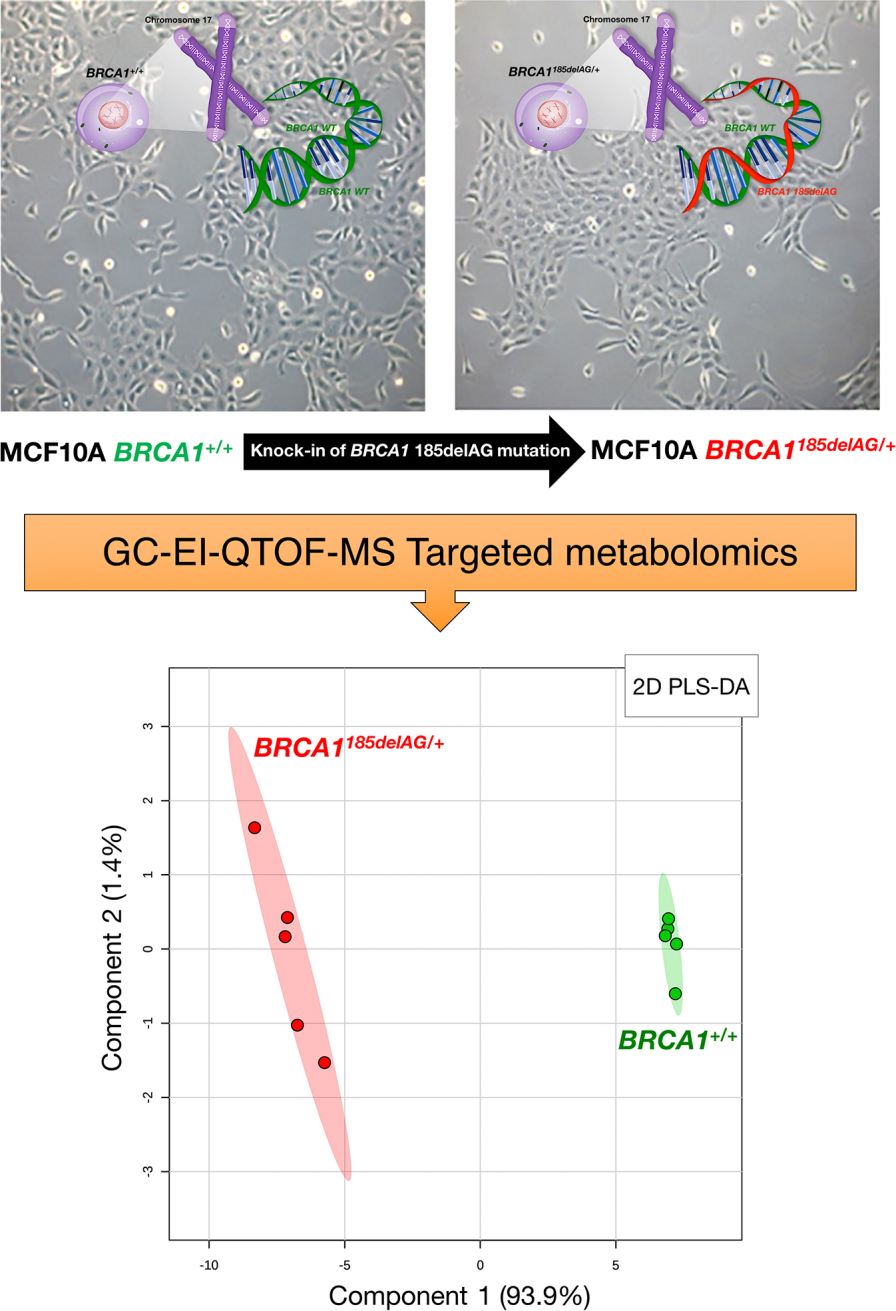
Metabolomic profiling of *BRCA1* haploinsufficient breast epithelial cells *Top.* Inactivating mutation of a single *BRCA1* allele leads to haploinsufficiency, which results in genomic instability in the spontaneously immortalized MCF10A cell line [14]. *Bottom.* 2D PLS-DA models of the GC-EI-QTOF-MS-based metabolomic profiling for the *BRCA1*^185delAG/+^ haploinsufficient and *BRCA1*^+/+^ wild-type cell groups.

To model *BRCA1* haploinsufficiency, we employed a well-defined experimental system using MCF10A breast epithelial cells, a spontaneously immortal and genetically stable basal-like model with wild-type *p53*. A common pathogenic mutation, *185delAG*, a 2-bp deletion in the coding region close to the N-terminus, was introduced into *BRCA1* by gene targeting [14; Figure 1, *top*]. This heterozygous mutation leads to haploinsufficiency and genomic instability in MCF10A cells, and therefore accurately mimics the cell-autonomous consequences of one-hit *BRCA1* inactivation. Metabolite-based clustering obtained by partial least squares-discriminant analysis (PLS-DA) model revealed a clear and significant separation between *BRCA1*^185delAG/+^ heterozygous cells and parental *BRCA1*^+/+^ cells in two-dimensional (2D) score plots (Figure 1, *bottom*).

**Figure 2:**
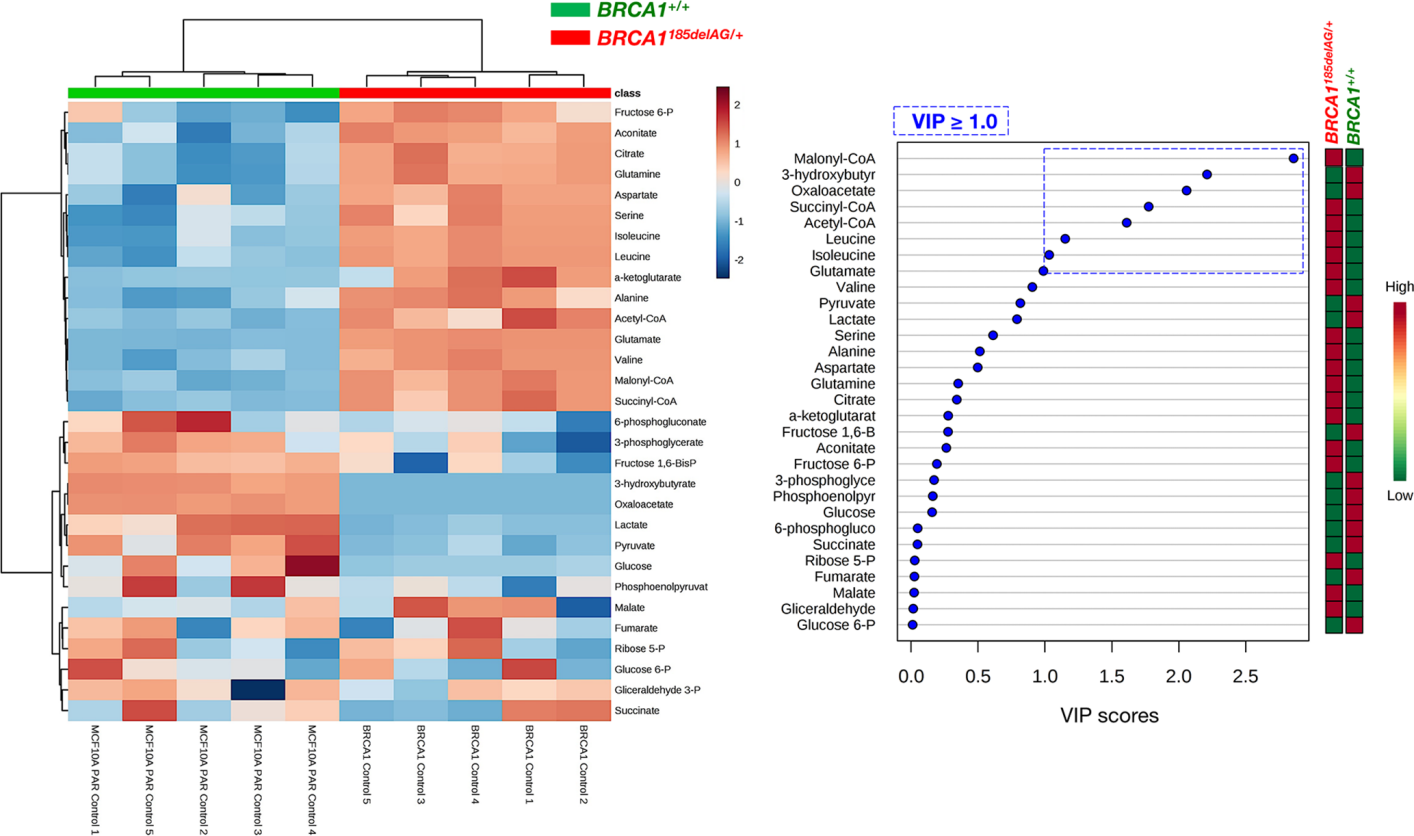
Inactivating mutation of a single *BRCA1* allele leads to metabolic reprogramming towards an anabolic phenotype *Left.* Heatmap visualization and hierarchical clustering analysis with MetaboAnalyst's data annotation tool for the *BRCA1*^185delAG/+^ haploinsufficient and *BRCA1*^+/+^ wild-type cell groups. Rows: metabolites; columns: samples; color key indicates metabolite expression value (blue: lowest; red: highest). *Right.* VIP rank-score of quantified metabolites in *BRCA1*^185delAG/+^haploinsufficient and *BRCA1*^+/+^ wild-type cell groups. Dashed blue box indicates metabolites that achieved VIP scores above 1.0.

**Figure 3:**
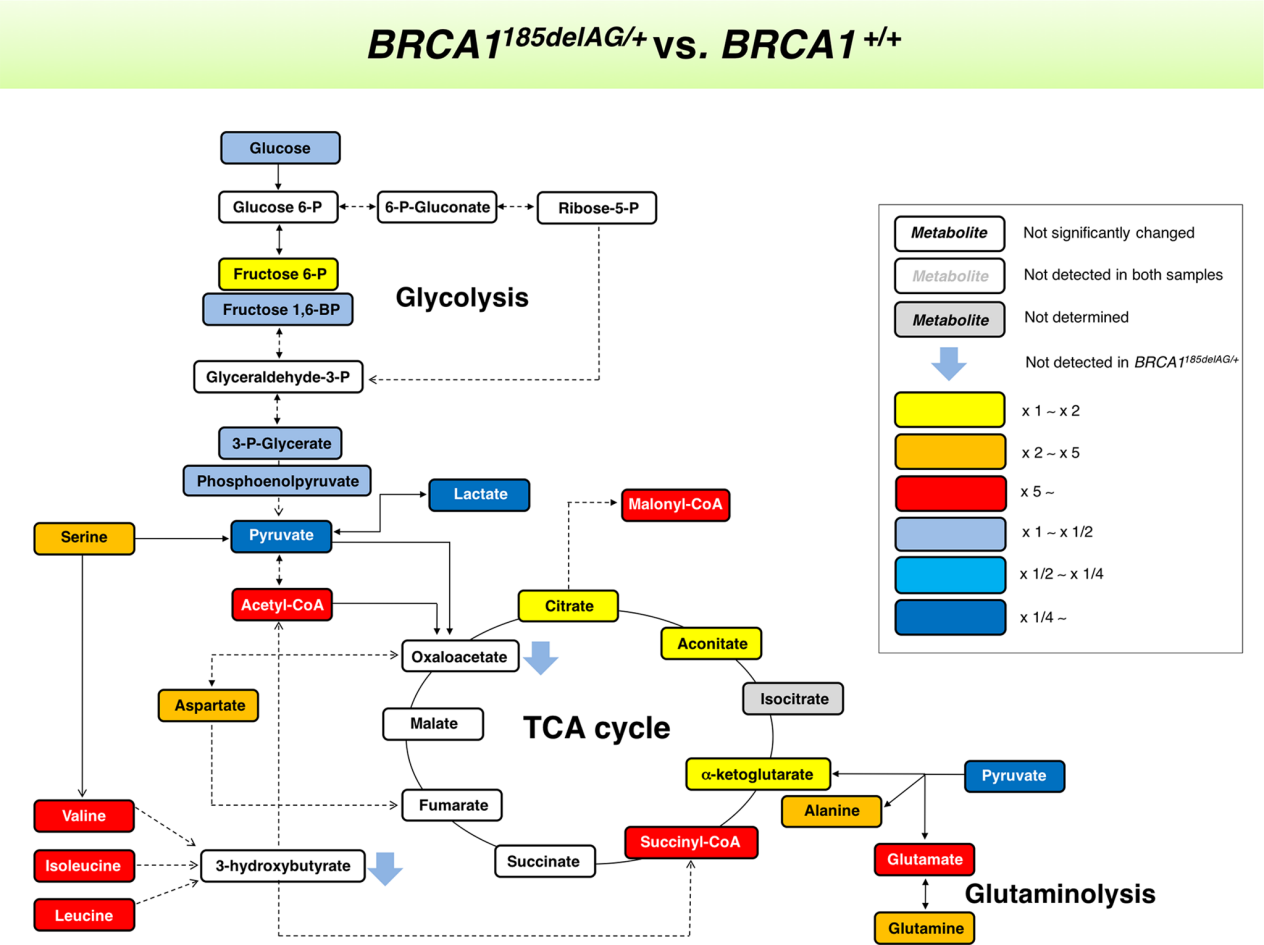
*BRCA1* “one-hit”-induced metabolic changes in breast epithelial cells Metabolites in *BRCA1*^185delAG/+^ haploinsufficient cells were extracted and quantitatively analyzed by GC-EI-QTOF-MS and compared with metabolites from control *BRCA1*^+/+^ isogenic parental cells. Significantly increased and decreased metabolites are shown using yellow-red and light blue-dark blue color scales, respectively.

Heatmap visualization, commonly used for unsupervised clustering, revealed distinct segregation of metabolites in *BRCA1*^185delAG/+^ and *BRCA1*^+/+^ groups (Figure 2, *left*), pointing to a metabolic signature associated with the human *BRCA1* mutation carrier state. In general, cellular metabolites related to a highly anabolic (biosynthetic) phenotype were more overrepresented in *BRCA1*^185delAG/+^ cells than in *BRCA1*^+/+^ cells, which significantly upregulated metabolites related to an oxidative phenotype (Figure 3). Accordingly, when the distinct metabolites between groups were selected with the criterion of variable importance in the projection (VIP ≥ 1) in the PLS-DA model, several coenzyme A (CoA) derivatives, such as acetyl-CoA, succinyl-CoA, and particularly malonyl-CoA, as well as the branched-chain amino acids (BCCAs) valine, isoleucine, and leucine, were significantly higher in *BRCA1*^185delAG/+^ cells than in *BRCA1*^+/+^ cells (Figure 2, *right*).

### The anti-diabetic biguanide metformin reduces tumor-initiating capacity of breast epithelial *BRCA1* one-hit cells

**Figure 4:**
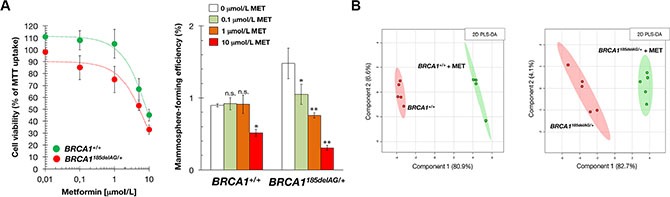
Treatment with the mitochondrial poison metformin suppresses mammosphere-initiating capacity of *BRCA1* haploinsufficient breast epithelial cells (**A**) *Left.* Relative cell viability of *BRCA1*^185delAG/+^ and *BRCA1*^+/+^ cells treated with graded concentrations of metformin. Cell viability was normalized to the condition with no metformin. Mean (*circles*) and SD (*bars*) were calculated from three biological replicates. *Right.* Seven-day MSFE of *BRCA1*^185delAG/+^ and *BRCA1*^+/+^ cells seeded in mammosphere medium in the absence or presence of graded concentrations of metformin and expressed as percentage mean (*columns*) ± SD (*bars*). Re-feeding of mammosphere cultures with metformin and/or sphere medium was performed on day 3 and 5. *P < 0.05; **P < 0.01 *versus* the corresponding controls. (**B**) Two-dimensional PLS-DA models of the GC-EI-QTOF-MS-based metabolomic profiling for the untreated and metformin-treated *BRCA1*^185delAG/+^ haploinsufficient and *BRCA1*^+/+^ wild-type cell groups (48 h treatment, 1 mmol/L metformin [MET]).

Because metformin has attracted interest as an antitumor agent in cancer prevention and treatment [39–53], we explored the possible impact of metformin treatment on metabolic rewiring induced by pathogenic mutation of a single *BRCA1* allele. Although both *BRCA1*^185delAG/+^ and *BRCA1*^+/+^ isogenic cells exhibited dose-dependent proliferative arrest in response to increasing concentrations of metformin as determined by MTT uptake, *BRCA1*^185delAG/+^ cells were more sensitive to the cytostatic effects of metformin than *BRCA1*^+/+^ parental cells (Figure 4A, *left*).

*BRCA1* is involved in regulating stemness and differentiation in stem and breast progenitor cells [54–57]. Accordingly, *BRCA1* haploinsufficiency increases three-dimensional clonal growth and proliferation of primary mammary epithelial cells from *BRCA1* mutation carriers. As one of the properties of stem/progenitor cells is their ability to survive, proliferate and differentiate under anchorage-independent conditions and generate spheroid structures [58], we tested the ability of *BRCA1*^185delAG/+^ and *BRCA1*^+/+^ cells to form tumorspheres (mammospheres) in suspension culture in the presence of metformin. Mammosphere forming-efficiency (MSFE) was calculated by dividing the number of sphere-like structures formed after 7 days (diameter > 50 μm) by the original number of cells seeded. A significantly higher number of *bona fide* mammospheres were formed from nontreated *BRCA1*^185delAG/+^ one-hit epithelial cells than nontreated *BRCA1*^+/+^ cells (Figure 4A, *right*), strongly suggesting that *BRCA1* haploinsufficiency led to a significant expansion of the stem cell-like population. This stronger spheroid formation capacity of *BRCA1*^185delAG/+^ cells was significantly and dose-dependently reduced by metformin, whereas baseline MSFE of *BRCA1*^+/+^ parental cells remained largely unresponsive to the drug (Figure 4A, *right*). Metformin-induced suppression of mammosphere formation (e.g., ≈50% reduction at 1 μmol/L metformin) was not attributed to non-specific toxic effects since cell viability in monolayer cultures remained as high as 75 ± 11% in the presence of an identical concentration of metformin (Figure 4A, *left*).

### Metformin suppresses mitochondrial-dependent production of biosynthetic intermediates in *BRCA1* haploinsufficient cells

We next explored whether the ability of metformin to reduce tumor-initiating capacity in *BRCA1* one- hit cells correlated with a capacity to alter the highly anabolic (biosynthetic) signature driven by *BRCA1* haploinsufficiency. PLS-DA models revealed a significant separation between untreated and metformin-treated breast epithelial cells regardless of their *BRCA1* status (Figure 4B). Thus, we speculated that the ability of metformin to suppress mammosphere formation in *BRCA1* one-hit cells related to its ability to specifically deactivate biosynthetic mitochondrial nodes in *BRCA1* one-hit cells but not in *BRCA1*^+/+^ cells.

**Figure 5:**
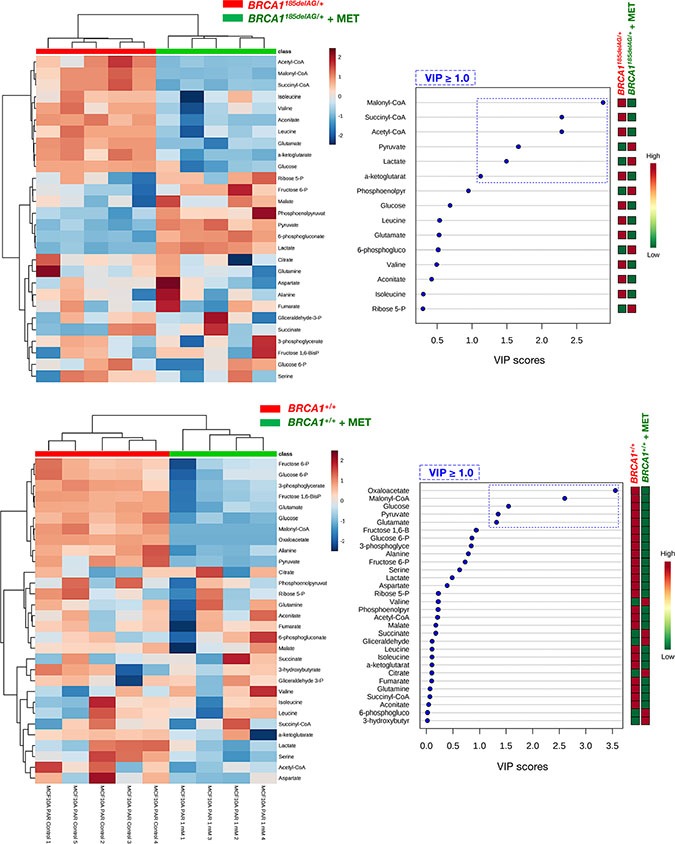
Metformin treatment specifically and significantly reverses the anabolic signature of *BRCA1* haploinsufficient breast epithelial cells *Left panels.* Heatmap visualization and hierarchical clustering analyses with MetaboAnalyst's data annotation tool for the untreated and metformin-treated (1 mmol/L MET; 48 h) *BRCA1*^185delAG/+^ haploinsufficient (*top*) and *BRCA1*^+/+^ wild-type (*bottom*) cell groups. Rows: metabolites; columns: samples; color key indicates metabolite expression value (blue: lowest; red: highest). *Right panels.* VIP rank-score of quantified metabolites in untreated and metformin-treated *BRCA1*^185delAG/+^ haploinsufficient (*top*) and *BRCA1*^+/+^ wild-type (*bottom*) cell groups. Dashed blue box indicates metabolites that achieved VIP scores above 1.0.

A general screen of the 30 selected metabolites using Heatmap visualization showed distinct segregation and revealed the ability of metformin to reverse the metabolomic signature and anabolic phenotype of mutant *BRCA1* one-hit cells by: a) shutting down mitochondria-driven generation of lipogenic precursors, and b) reducing the pool of BCAAs for protein synthesis and/or anaplerotic fueling of TCA cycle metabolism (Figure 5, *top*). When VIP scores ≥ 1 in the PLS-DA model were used to maximize the difference of metabolic profiles between untreated and metformin-treated *BRCA1*^185delAG/+^ groups, it was noteworthy that the subset of metabolites majorly impacted by metformin included the basic building blocks for the *de novo* synthesis of fatty acids malony-CoA and acetyl-CoA, the TCA metabolites succinyl-CoA and α-ketoglutarate, and the end-products of glycolysis lactate and pyruvate (Figure 5, *top*). A largely different metabolic rearrangement occurred after metformin treatment of *BRCA1*^+/+^ cells (Figure 5, *bottom*), strongly suggesting that metabolic responses to metformin-induced inhibition of complex I-dependent mitochondrial respiration depended on the intrinsic engagement of a given cell type to mitochondrial metabolism [59, 60].

**Figure 6:**
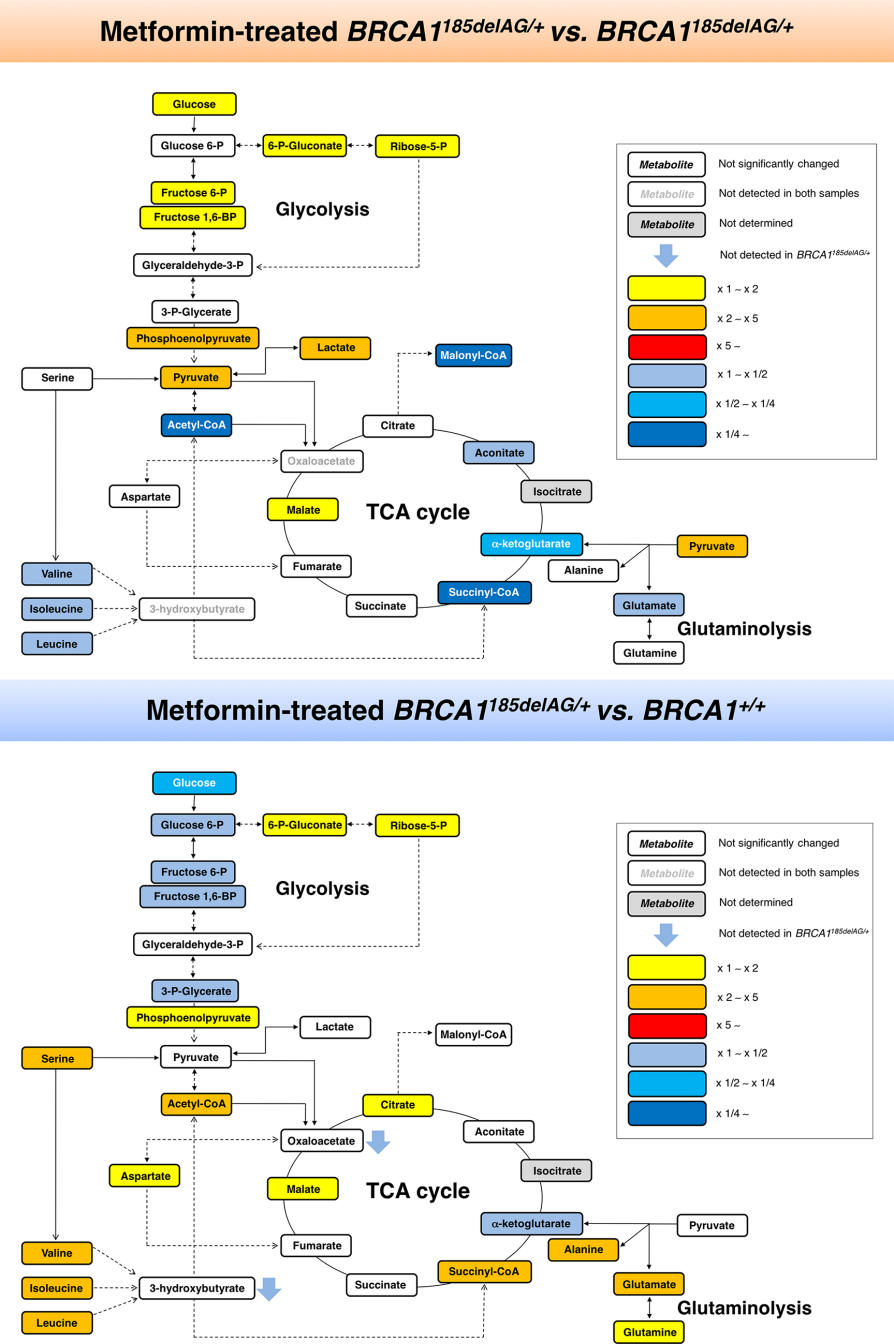
Metformin-induced metabolic changes in *BRCA1* haploinsufficient breast epithelial cells Metabolites in metformin (MET)-treated *BRCA1*^185delAG/+^ haploinsufficient cells were extracted and quantitatively analyzed by GC-EI-QTOF-MS and compared with metabolites from either untreated *BRCA1*^185delAG/+^ haploinsufficient cells (*top*) or untreated *BRCA1*^+/+^ isogenic parental cells (*bottom*). Significantly increased and decreased metabolites are shown using yellow-red and light blue-dark blue color scales, respectively.

Quantitative assessment of metabolite concentrations confirmed that treatment with metformin fully suppressed (> 90% reduction) the major accumulation (≈162-fold increase) of malonyl-CoA and acetyl-CoA (23-fold increase) induced by *BRCA1*^185delAG/+^ mutation (Table 1, Figure 6) Similarly, treatment with metformin reduced, by ≈90%, the 27-fold accumulation of succinyl-CoA and, by 60%, the levels of α-ketoglutarate in *BRCA1*^185delAG/+^ cells (Table 1). Although less dramatically, 1 mmol/L metformin reduced by up to 40% the 5-, 7- and 6-fold accumulation of the BCAAs valine, leucine, and isoleucine, respectively, in *BRCA1*^185delAG/+^ cells. Conversely, metformin treatment increased, by ≈4- and 5-fold, the intracellular levels of lactate and pyruvate in *BRCA1*^185delAG/+^ cells, respectively.

**Table 1:** Concentration (in μM/mg of protein ± SEM) and fold change of bioenergetics metabolites in parental *BRCA1*^+/+^ and their *BRAC1*^185delAG/+^ haploinsufficient derivatives

Metabolite		*BRCA1^+/+^*	*BRCA1^+/–^*	Fold-change [*BRCA1^+/^/BRCA1^+/+^*]
**Malonyl-CoA**	Control	0.0016 ± 0.0002 (1.0)	0.26 ± 0.05 (1.00)	[162.5]
	+ 1 mM MET	N.D.	0.01658 ± 0.0007 (0.06)	-
**Succinyl-CoA**	Control	0.012 ± 0.001 (1.0)	0.32 ± 0.07 (1.0)	[26.7]
	+ 1 mM MET	0.012 ± 0.002 (1.0)	0.035 ± 0.003 (0.1)	
**Acetyl-CoA**	Control	0.015 ± 0.001 (1.0)	0.34 ± 0.09 (1.00)	[23]
	+ 1 mM MET	0.0122 ± 0.0006 (0.8)	0.032 ± 0.001 (0.09)	
**3-Hydroxybutyrate**	Control	5.2 ± 0.4 (1.0)	N.D.	-
	+ 1 mM MET	5.2 ± 0.2 (1.0)	N.D.	-
**3-Phosphoglycerate**	Control	2.9 ± 0.2 (1.0)	2.2 ± 0.2 (1.0)	[0.8]
	+ 1 mM MET	1.2 ± 0.1 (0.4)	2.1 ± 0.5 (1.0)	
**6-Phosphogluconate**	Control	6.9 ± 0.2 (1.0)	6.3 ± 0.1 (1.0)	[0.9]
	+ 1 mM MET	7.2 ± 0.8 (1.0)	10.1 ± 0.2 (1.6)	
**Aconitate**	Control	0.31 ± 0.02 (1.0)	0.49 ± 0.01 (1.0)	[1.6]
	+ 1 mM MET	0.31 ± 0.05 (1.0)	0.34 ± 0.01 (0.7)	
**α-ketoglutarate**	Control	0.625 ± 0.003 (1.0)	1.04 ± 0.09 (1.0)	[1.7]
	+ 1 mM MET	0.56 ± 0.05 (0.9)	0.37 ± 0.04 (0.4)	
**Alanine**	Control	112 ± 11 (1.0)	277 ± 21 (1.0)	[2.5]
	+ 1 mM MET	49 ± 4 (0.4)	261 ± 19 (0.9)	
**Aspartate**	Control	112 ± 19 (1.0)	258 ± 11 (1.0)	[2.3]
	+ 1 mM MET	72 ± 6 (0.6)	224 ± 18 (0.9)	
**Citrate**	Control	7.0 ± 0.5 (1.0)	12.7 ± 0.4 (1.0)	[1.8]
	+ 1 mM MET	7.9 ± 0.9 (1.1)	11.6 ± 0.7 (0.9)	
**Fructose 1,6-bisphosphate**	Control	0.55 ± 0.01 (1.0)	0.35 ± 0.05 (1.0)	[0.6]
	+ 1 mM MET	0.21 ± 0.03 (0.4)	0.44 ± 0.07 (1.3)	
**Fructose 6-phosphate**	Control	1.24 ± 0.09 (1.0)	1.73 ± 0.06 (1.0)	[1.4]
	+ 1 mM MET	0.6 ± 0.1 (0.5)	2.03 ± 0.08 (1.2)	
**Fumarate**	Control	4.0 ± 0.2 (1.0)	3.8 ± 0.2 (1.0)	[1.0]
	+ 1 mM MET	3.7 ± 0.5 (1.0)	4.0 ± 0.5 (1.0)	
**Glyceraldehyde 3-phosphate**	Control	2.6 ± 0.4 (1.0)	2.6 ± 0.2 (1.0)	[1.0]
	+ 1 mM MET	2.9 ± 0.4 (1.1)	2.6 ± 0.5 (1.0)	
**Glucose**	Control	7.6 ± 0.7 (1.0)	5.66 ± 0.04 (1.0)	[0.7]
	+ 1 mM MET	1.6 ± 0.2 (0.2)	3.2 ± 0.5 (0.6)	
**Glucose 6-phosphate**	Control	2.7 ± 0.3 (1.0)	2.6 ± 0.3 (1.0)	[1.0]
	+ 1 mM MET	1.2 ± 0.2 (0.4)	2.3 ± 0.4 (0.9)	
**Glutamate**	Control	21.8 ± 0.4 (1.0)	126 ± 2 (1.0)	[5.8]
	+ 1 mM MET	5.9 ± 0.9 (0.3)	78 ± 4 (0.6)	
**Glutamine**	Control	7.5 ± 0.4 (1.0)	13.9 ± 0.4 (1.0)	[1.8]
	+ 1 mM MET	7.4 ± 1.5 (1.0)	13.4 ± 0.3 (1.0)	
**Isoleucine**	Control	15 ± 3 (1.0)	87 ± 4 (1.0)	[5.8]
	+ 1 mM MET	13 ± 2 (0.9)	68 ± 7.4 (0.8)	
**Lactate**	Control	1638 ± 293 (1.0)	373 ± 16 (1.0)	[0.2]
	+ 1 mM MET	944 ± 125 (0.6)	1490 ± 115 (4.0)	
**Leucine**	Control	35 ± 7 (1.0)	260 ± 12 (1.0)	[7.4]
	+ 1 mM MET	32 ± 6 (0.9)	161 ± 14 (0.6)	
**Malate**	Control	2.06 ± 0.05 (1.0)	2.2 ± 0.1 (1.0)	[1.1]
	+ 1 mM MET	1.834 ± 0.3 (0.9)	2.7 ± 0.2 (1.2)	
**Oxaloacetate**	Control	39 ± 2 (1.0)	N.D.	-
	+ 1 mM MET	N.D.	N.D.	
**Phosphoenolpyruvate**	Control	2.1 ± 0.3 (1.0)	1.5 ± 0.1 (1.0)	[0.7]
	+ 1 mM MET	1.7 ± 0.3 (0.8)	3.0 ± 0.3 (2.0)	
**Pyruvate**	Control	35 ± 6 (1.0)	7.7 ± 0.7 (1.0)	[0.2]
	+ 1 mM MET	8.0 ± 0.3 (0.2)	36 ± 4 (4.7)	
**Ribose 5-phosphate**	Control	1.8 ± 0.3 (1.0)	1.9 ± 0.2 (1.0)	[1.0]
	+ 1 mM MET	1.5 ± 0.3 (0.8)	2.5 ± 0.2 (1.3)	
**Serine**	Control	118 ± 18 (1.0)	338 ± 28 (1.0)	[2.9]
	+ 1 mM MET	59 ± 5 (0.5)	301 ± 27 (0.9)	
**Succinate**	Control	24 ± 3 (1.0)	22 ± 4 (1.0)	[0.9]
	+ 1 mM MET	29 ± 6 (1.2)	24 ± 4 (1.1)	
**Valine**	Control	15 ± 1 (1.0)	74 ± 4 (1.0)	[4.9]
	+ 1 mM MET	19 ± 3 (1.3)	48 ± 5 (0.6)	

Decimals were set according to the first significant digit of the measured SEM.

**Figure 7:**
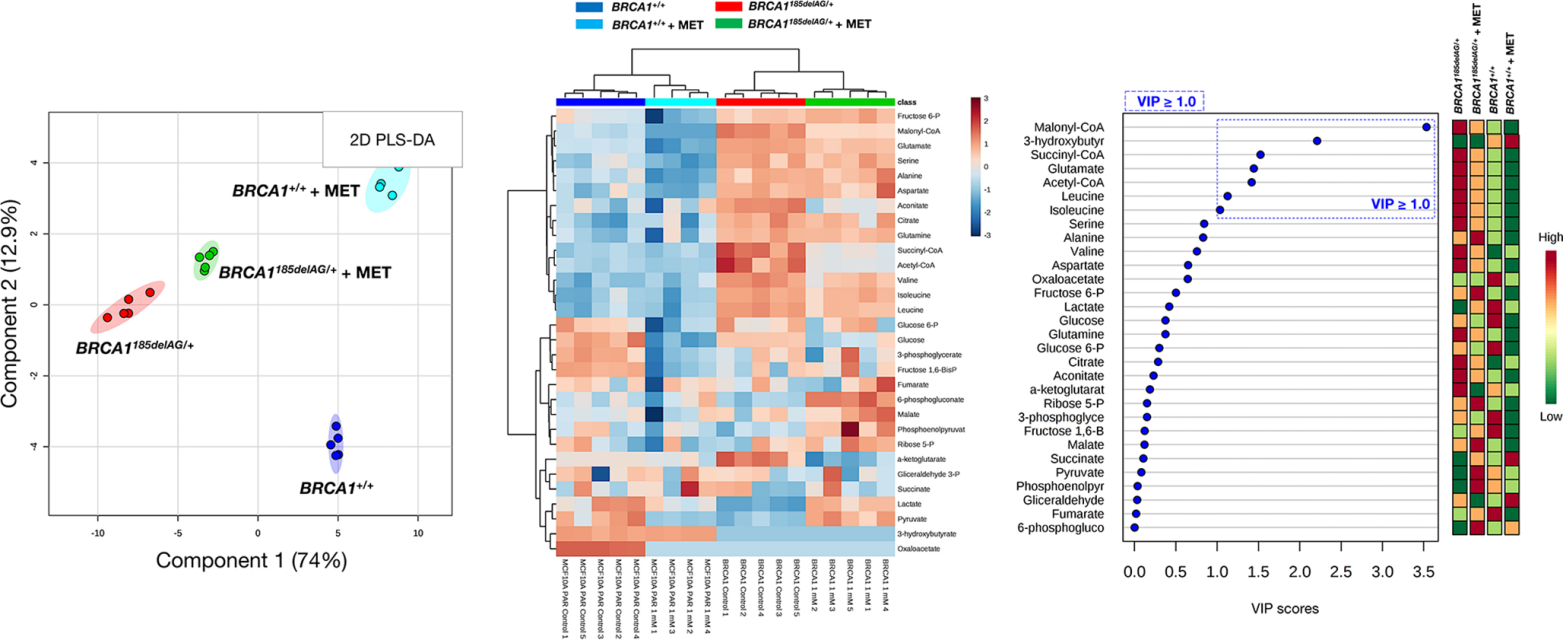
Metformin treatment generates a new metabolomic signature in *BRCA1* haploinsufficient breast epithelial cells *Left.* Two-dimensional PLS-DA models to view the separation of the 4 groups (untreated *BRCA1*^185delAG/+^ haploinsufficient cells, metformin [MET]-treated *BRCA1*^185delAG/+^ haploinsufficient cells, untreated *BRCA1*^+/+^ wild-type cells, and metformin-treated *BRCA1*^+/+^ wild-type cells) following GC-EI-QTOF-MS-based metabolomic profiling. *Middle.* Heatmap to view the agglomerative hierarchical clustering of the 4 groups analyzed with MetaboAnalyst's data annotation tool. Rows: metabolites; columns: samples; color key indicates metabolite expression value (blue: lowest; red: highest). *Right.* VIP rank-score of quantified metabolites in the 4 groups. Dashed blue box indicates metabolites that achieved VIP scores above 1.0.

Although the key carbon donor for *de novo* fatty acid synthesis, malonyl-CoA, remained one of the most affected metabolites in metformin-treated *BRCA1*^+/+^ cells, it was noteworthy that this treatment triggered a largely different metabolic rearrangement when compared with *BRCA1*^185delAG/+^ cells (Figure 7). Moreover, although metformin treatment of *BRCA1*^185delAG/+^ cells failed to restore the metabolic portrait originally exhibited by *BRCA1*^+/+^ cells, a global assessment of untreated *BRCA1*^+/+^, metformin-treated *BRCA1*^+/+^, untreated *BRCA1*^185delAG/+^, and metformin-treated *BRCA1*^185delAG/+^ classes based on the VIP parameter once again revealed that the CoA derivatives malonyl-CoA, succinyl-CoA, and acetyl-CoA, and the BCCAs leucine and isoleucine were the key metabolites responsible for separation of all the metabolomic classes (Figure 7).

### Metformin differentially impacts the capacity of normal-like and BRCA1 haploinsufficient cells to oxidize glucose and glutamine

**Figure 8:**
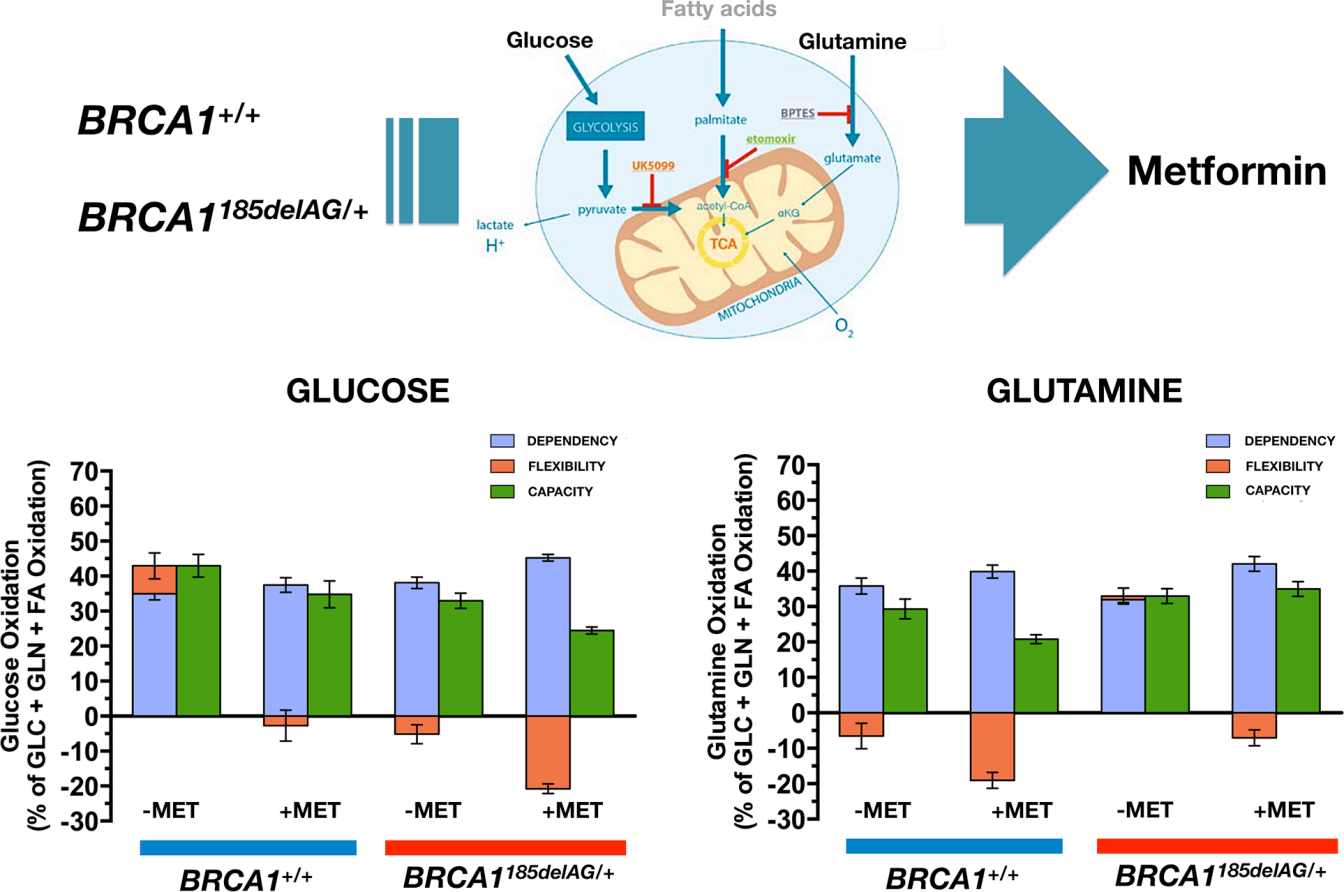
Metformin treatment differentially impacts mitochondrial fuel usage in *BRCA1* haploinsufficient breast epithelial cells Figure shows the dependency, capacity, and flexibility of *BRCA1*^185delAG/+^ haploinsufficient and *BRCA1*^+/+^ wild-type cells to oxidize the mitochondrial fuels glucose (left) and glutamine (right) in the absence or presence of metformin.

Because mitochondrial access to different fuels such as glucose and glutamine significantly impacts a variety of biological processes, we assessed whether *BRCA1*^+/+^ and *BRCA1*^185delAG/+^ breast epithelial cells might differentially adjust fuel oxidation to match nutrient availability while meeting energy demand. Taking advantage of the Seahorse XF Mito Fuel Flex Test, a comprehensive method for measuring mitochondrial fuel usage in live cells, we measured the dependency (i.e., the requirement for a specific fuel to meet metabolic demand), capacity (i.e., the ability to use a specific fuel to meet energy demand), and flexibility (i.e., the difference between the amount they need and the amount they could use) of *BRCA1*^+/+^ and *BRCA1*^185delAG/+^ breast epithelial cells to oxidize glucose and glutamine when pre-challenged with metformin (5 μmol/L, 24 h). *BRCA1*^185delAG/+^ breast epithelial cells exhibited a significantly lower capacity to use and increase the oxidation of glucose when trying to compensate for BPTES- and etomoxir-induced inhibition of alternative fuel pathways (i.e., glutamine oxidation and long chain fatty acid oxidation, respectively), a mitochondrial fuel usage that was further exacerbated when *BRCA1* haploinsufficient cells were pre-exposed to metformin (Figure 8, *left*). Conversely, *BRCA1*^185delAG/+^ breast epithelial cells exhibited a significantly higher capacity to use and increase the oxidation of glutamine when trying to compensate for UK5099 and etomoxir-induced inhibition of alternative fuel pathways (i.e., glucose oxidation and long chain fatty acid oxidation, respectively), a mitochondrial fuel usage that was further exacerbated when *BRCA1* haploinsufficient cells were pre-exposed to metformin (Figure 8, *right*).

### Metformin reverts the activation of mTOR-related signaling in BRCA1 haploinsufficient cells

**Figure 9:**
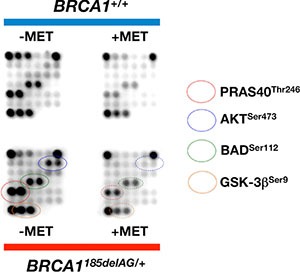
Metformin suppresses *BRCA1* haploinsufficiency-driven activation of the AKT/mTOR pathway Figure shows representative chemiluminiscent array images of the PathScan Intracellular Signaling array kit revealing various phosphorylated signaling nodes in *BRCA1*^185delAG/+^ haploinsufficient and *BRCA1*^+/+^ wild-type cells cultured in the absence or presence of metformin.

To explore whether the apparent anabolic reprogramming of *BRCA1* haploinsufficient cells involved the activation of signal transduction mechanisms linked to mTOR, we took advantage of commercially available slide-based antibody arrays for the simultaneous assessment of 18 well-characterized intracellular signaling molecules (Figure 9). *BRCA1*^185delAG/+^ breast epithelial cells exhibited a noteworthy hyperactivation of the oncogenic protein AKT, a well-known controller of protein synthesis at the level of translation initiation and ribosome biogenesis [61, 62], when compared to *BRCA1*^+/+^ parental cells. The AKT/mTOR-related activation of protein synthesis driven by *BRCA1* haploinsufficiency was confirmed by the hyperphosphorylation of the proline-rich AKT substrate of 50 kDa (PRAS40) at Thr246 by AKT, which is known to act at the intersection of the AKT and mTOR-mediated signaling by relieving PRAS40 inhibition of the mTOR complex 1 (mTORC1) [63–65]. Interestingly, a 48 h treatment with metformin fully suppressed the activation of the AKT/mTOR-signaling network in *BRCA1* haploinsufficient cells (Figure 9). Indeed, metformin-treated *BRCA1*^185delAG/+^ breast epithelial cells treatment failed to phosphorylate the pro-apoptotic protein BAD at Ser112 and the multifunctional kinase GSK-3β at Ser9, an AKT-driven signaling that is known to inhibit BAD and GSK-3β activities to promote cell survival [66, 67].

## DISCUSSION

In addition to oncogenic mutations, a shift towards cancer-like metabolic changes including increased aerobic utilization of glucose to produce lactate mediated by glycolysis (Warburg effect) [68] and enhanced mitochondrial utilization of glucose and glutamine to sustain anabolic processes [69–78], might pre-set an idoneous metabolic stage for later mutations to drive tumorigenesis [31–33]. Here, we present evidence that inheritance of a mutated *BRCA1* allele might expedite the establishment of a metabolic state compatible and “permissive” with the elevated genomic instability found in breast epithelial cells of women carrying a non-functional copy of *BRCA1*.

To explore whether the one-hit event might be sufficient to shorten the time required for breast epithelial cells to become “pre-equipped” to support the metabolically-demanding process of tumor formation, we utilized a GC-EI-QTOF-MS-based metabolomic approach to examine whether metabolites involved in energy metabolism, including glycolysis, the TCA cycle, and pathways involved in metabolism of lipids, amino acids, and pentose phosphates were altered in response to “one-hit” *BRCA1*. Our present findings showing a dramatic accumulation of the acyl-CoA derivatives succinyl-CoA, acetyl-CoA, and malonyl-CoA accompanied by high levels of BCAAs in *BRCA1*^185delAG/+^ heterozygous cells, strongly suggest that germline mutation in a *BRCA1* allele is sufficient to phenocopy multiple *bona fide* oncogenic pathways to stimulate glucose- and glutamine-derived carbon atom flux, promote TCA metabolism and lipogenesis, and coordinate carbon and nitrogen metabolism across amino acids to likely stimulate mTOR-driven protein synthesis [73–86].

Compounds that interfere with early stages of cancer formation are particularly appropriate for mutation carriers and evidence indicates that risk reduction strategies focused on patients harboring germ-line *BRCA1* mutations are effective [87–89]. In this regard, our present results established that pharmacological ablation of *BRCA1* mutation-driven hyperactivation of mitochondrial-dependent biosynthetic activity was sufficient to reduce the tumor-initiating capacity of breast epithelial *BRCA1* one-hit cells in mammosphere assays. Our findings that breast epithelial *BRCA1* one-hit cells produced a greater number of mammospheres than controls extend previous studies showing that breast epithelial cells from *BRCA1* mutation carriers form cell colonies in non-adherent conditions with greater efficiency than control epithelial cells [54–57]. Importantly, we could establish a causal, functional relationship between the metabolic rewiring of *BRCA1* one-hit cells and their increased mammosphere-forming capacity, a surrogate measure of CSCs. Thus, we concluded that metformin treatment reduced the tumor-initiating (self-renewal) capacity of breast epithelial *BRCA1* one-hit cells, whereas *BRCA1* wild-type cells were mostly resistant at similar doses, which is indicative of a wide therapeutic index. Remarkably, the dissimilar responses of *BRCA1* one-hit cells and *BRCA1* wild-type cells to metformin in terms of stemness properties directly correlated with a more drastic and specific rearrangement of the anabolic phenotype in *BRCA1* one-hit cells, supporting the notion that cells with high mitochondrial metabolism are more metabolically responsive to metformin [59, 60].

Mechanistically, our results indicated that metformin targeted the tumorigenic capacity of one-hit *BRCA1* cells by starving mitochondria of the necessary metabolic intermediates required for anabolic metabolism. Our TCA metabolite screen revealed that metformin treatment significantly reduced the total abundance of α-ketoglutarate, accompanied by the suppression of malonyl-CoA generation, the major building block for lipid biosynthesis. Although it might be argued that the ability of metformin to interfere with the anaplerotic entry of glutamine into the TCA cycle can explain the increased sensitivity of *BRCA1* one-hit cells to its anti-tumor initiating capacity, it should be also acknowledged that metformin reduced glucose-derived acetyl-CoA entry into mitochondrial metabolism in *BRCA1* one-hit cells more dramatically than in *BRCA1* wild-type cells. Indeed, *BRCA1* one-hit cells treated with metformin had an increased fraction of total malate within the TCA cycle while also increasing the intracellular concentration of pyruvate and lactate. The reduced usage of pyruvate by the TCA cycle accompanied by increased generation of lactate clearly indicated that metformin treatment diverted pyruvate away from mitochondrial metabolism by increasing glycolysis activity in *BRCA1* one-hit cells. Therefore, inhibition of complex I-dependent mitochondrial respiration by metformin in turn inhibits the overactive TCA cycle in *BRCA1* one-hit cells, which accept less glucose carbon, favoring lactic acid production. In addition, metformin treatment also impedes the glutamine contribution to anaplerotic flux into the TCA cycle, and the previously argued adaptive response to metformin involving a shift in α-ketoglutarate metabolism towards reductive carboxylation, failed to replenish the high-levels of lipogenic acetyl-CoA induced by *BRCA1* haploinsufficiency. Moreover, because valine and isoleucine contain carbons that form succinyl-CoA, whereas the degradation of isoleucine and leucine forms acetyl-CoA, the ability of metformin to reduce the pool of BCAAs, which comprises almost 25% of the content of the average protein, might impede *BRCA1* one-hit cells to meet their elevated demand for alanine and glutamine and protein synthesis by impairing BCCA-driven activation of mTOR and TCA fueling.

**Figure 10:**
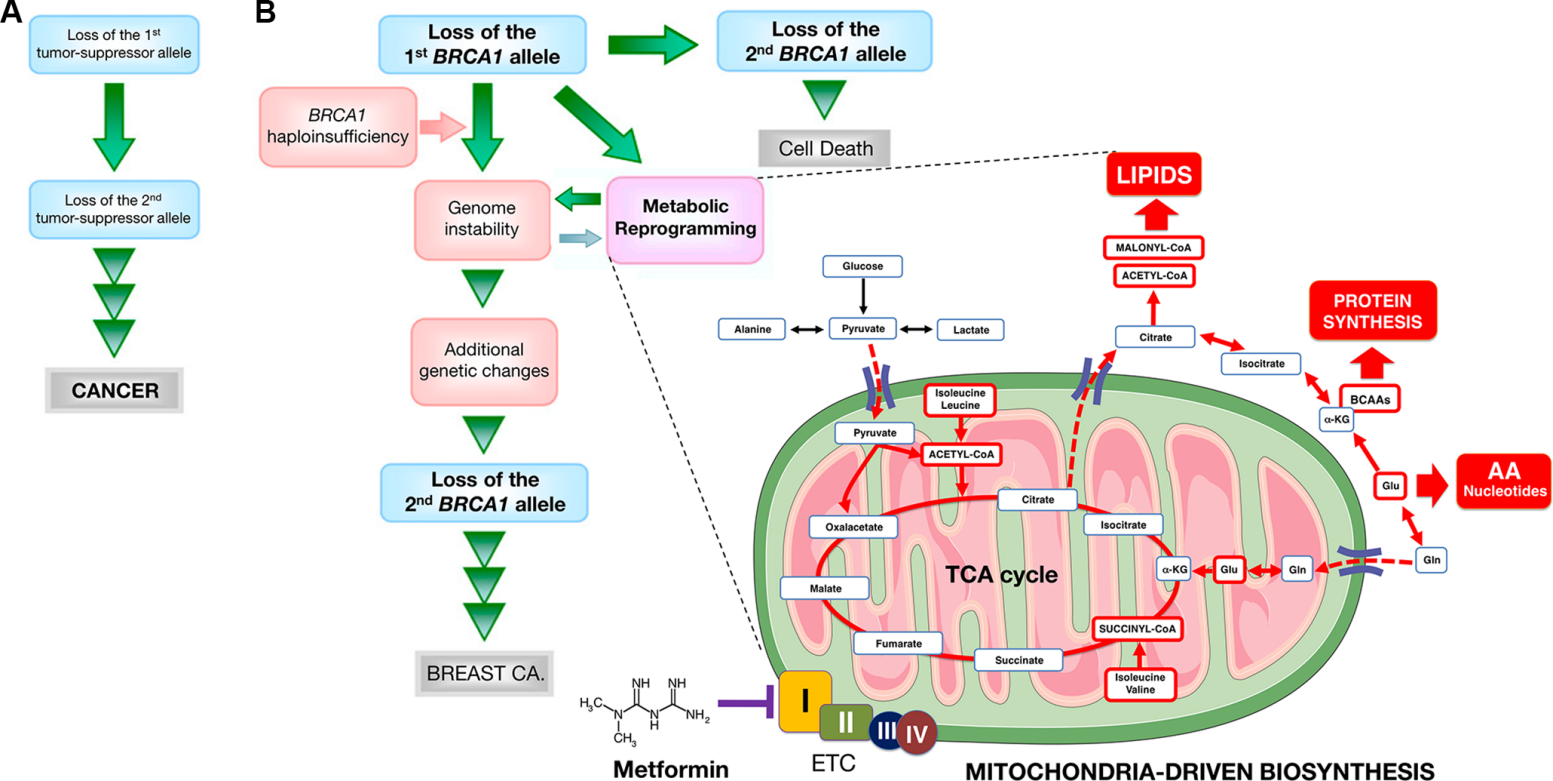
Mitochondrial-dependent biosynthesis: a therapeutically targetable feature of *BRCA1*-driven breast carcinogenesis A schematic overview of the concept in the present study. (**A**) Original “two-hit” theory for the incidence of familial cancers proposed by Knudson [5]. (**B**) A modified two-hit model for *BRCA1*-mediated breast carcinogenesis suggested by the present study. Because *BRCA1*-null cells seem to require additional genetic changes for survival, *BRCA1*-mediated breast carcinogenesis may be considered in a distinct manner from other familial cancer syndromes that follow the two-hit theory, in which consecutive deletion of two alleles accelerates tumorigenesis. Inactivating mutations of a single *BRCA1* allele leads to haploinsufficiency, which results in genomic instability in human breast epithelial cells. It has been suggested that genomic instability resulting from *BRCA1* haploinsufficiency is an early but not sufficient step for *BRCA1*-mediated breast carcinogenesis by allowing the accumulation of additional genetic changes that enable cancer progenitor cells to evade cell death that would otherwise occur upon loss of the remaining wild-type *BRCA1* allele. We propose that *BRCA1* haploinsufficiency is sufficient to produce significant and chronic changes in cellular metabolism by promoting a highly anabolic phenotype compatible and “permissive” with the elevated genomic instability present in *BRCA1* heterozygous cells. If *BRCA1* haploinsufficiency is exclusive to specific tissue types, such as breast epithelia, metabolic reprogramming towards mitochondrial-dependent biosynthesis in normal breast epithelial cells may provide a novel explanation for the restricted tissue distribution of *BRCA1* carcinogenesis and could be potentially exploited to develop prophylactic therapies for *BRCA1*-related cancers. The anti-diabetic mitochondrial poison metformin inhibits complex I of the electron transport chain (ETC) [97, 98], which drastically restricts the production of mitochondrial-dependent biosynthetic intermediates by reducing the anaplerotic flux of glucose, glutamine, and likely BCCAs into the TCA cycle. The ability of metformin to restrict the production of mitochondrial-dependent biosynthetic intermediates while depleting the pool of mTOR activating amino acids (e.g., leucine) might open a new avenue for “mitochondrial starvation” chemopreventive strategies in *BRCA1* carriers. (AA: amino acids; Gln: glutamine; Glu: glutamate; α-KG: alpha-ketoglutarate).

Tremendous progress has been made in the last 20 years in understanding how monoallelic *BRCA1* loss leads to defects in DNA damage response, chromatin organization, gene transcription, cell division, and protein and genome stability [90–92]. Nevertheless, it remains enigmatic why a mutation in a single copy of *BRCA1* leads to rapid tumor onset in a very strict tissue- and cell-type-specific manner. Our present results identify an inherited form of metabolic reprogramming and provide a conceivable metabolic basis for the rapid cell- and tissue-specific predisposition of breast cancer development associated with *BRCA1* haploinsufficiency. The metabolomic signature distinguishing wild-type from *BRCA1* haploinsufficient but otherwise isogenic breast epithelial cells reveals that a constitutively enhanced activation of mitochondrial-dependent biosynthesis becomes activated to sustain anabolic processes in mutant *BRCA1* cells. Our findings add a new dimension to Sinclair's original proposal of geroncogenesis [31], which states that normal decline in oxidative mitochondrial metabolism during aging constitutes an early and important “hit” that drives tumorigenesis. Our findings experimentally support our recent proposal that women carrying mutations in *BRCA1* might undergo a process of “accelerated geroncogenesis” in mammary epithelia [93]. The unanticipated ability of one germline *BRCA1* hit to reprogram breast epithelial host metabolism towards increased anabolism should broaden our understanding of “gatekeeper” or “caretaker” tumor-suppressor genes as well as improving our knowledge of the ultimate molecular requirements for *BRCA1*-driven neoplastic transformation in a tissue- and cell-type-specific manner. Although heterozygous *BRCA1* inactivation itself might be insufficient for complete breast carcinogenesis, the acceleration of the rate at which an “anabolic threshold” would permit the survival and expansion of genomically altered breast epithelial cells might operate as a true oncogenic event of *BRCA1* haploinsufficiency-driven breast carcinogenesis (Figure 10). Further studies are warranted to determine the ultimate role of *BRCA1* haploinsufficiency-driven metabolic reprogramming in the discrete evolutionary pathways occurring in *BRCA1-*associated breast tumors [94] and whether a similar metabolic facet should be contemplated when assessing the pathophysiology, consequences and therapeutic value of other well-known oncosuppressors.

Because *BRCA1* haploinsufficient cells display phenotypic alterations in cell-surface marker expression [56, 95] despite their normal histological appearance [56], metabolic rewiring of the breast epithelium towards increased anabolism can be employed to provide novel metabolic biomarkers in women bearing pathogenic germline *BRCA1* mutations. Moreover, the fact that *BRCA1* haploinsufficiency might accelerate the geroncogenic trait that naturally occurs in the breast epithelium also provides a rationale for new preventive and therapeutic strategies based on drugs or energetic behavior tools aimed to halt the breast tissue- and epithelial cell-specific metabolic reprogramming that might occur in *BRCA1* carriers. Our results indicate that metformin can limit the enhanced tumor-initiating capacity of mutant *BRCA1* one-hit cells by starving them of the biosynthetic intermediates required for an efficient anabolic cell growth and survival. Together, our data support a recently proposed “substrate limitation” model of metformin action [59, 60], in which metformin restricts the production of mitochondrial-dependent biosynthetic intermediates by reducing the anaplerotic flux of glucose, glutamine, and likely BCCAs, into the TCA cycle, leading to depletion of acetyl-CoA and malonyl-CoA required for *de novo* lipid biosynthesis and inhibition of mTOR-driven protein synthesis in anabolism-addicted *BRCA1* haploinsufficient cells (Figure 10). These observations can be exploited to develop new metformin-based “starvation” strategies as chemopreventive and treatment options in *BRCA1* carriers.

## MATERIALS AND METHODS

### Cell lines

Human *BRCA1* (185delAG/+) MCF10A cells with heterozygous knock-in of a 2-bp deletion of *BRCA1* resulting in a premature termination codon at position 39 and MCF10A isogenic parental cells were obtained from Horizon Discovery Ltd., Cambridge, UK (Cat# HD 101–018 and HD PAR-058, respectively). Cells were routinely grown in DMEM/F-12 (Gibco, Life Technologies, Paisley, UK) including 2.5 mmol/L L-glutamine, and 15 mmol/L HEPES, supplemented with 5% horse serum (HS), 10 μg/ mL insulin, 20 ng/mL hEGF, 0.5 μg/mL hydrocortisone and 0.1 μg/ mL cholera toxin. Cells were cultured as a monolayer at 37°C in a humidified atmosphere with 5% CO_2_. Cells were passaged every 3–5 days and split at 80–90% confluency, approximately 1:6–1:10.

### Targeted Metabolomics

Measurements of metabolites obtained from heterozygous *BRCA1*^185delAG/+^ and *BRCA1*^+/+^ isogenic parental cells cultured in the absence or presence of 1 μmol/L metformin (48 h) were performed by employing a previously described simple and quantitative method based on gas chromatography coupled to quadrupole-time of flight mass spectrometry and an electron ionization interface (GC-EI-QTOF-MS) [37, 38].

### Data analysis

Raw data were processed and compounds were detected and quantified using the Qualitative and Quantitative Analysis B.06.00 software (Agilent Technologies), respectively. MetaboAnalyst 3.0 (http://www.metaboanalyst.ca/) was used to generate scores/loading plots and Heatmaps [96].

### Cell viability assays

The cell viability effects of metformin were determined using the standard colorimetric MTT reduction assay. For each treatment, cell viability was evaluated as a percentage using the following equation: (OD_570_ of the treated sample/OD_570_ of the untreated sample)×100.

### Mammosphere culture and mammosphere-forming efficiency

Single cell suspensions of *BRCA1*^185delAG/+^ and *BRCA1*^+/+^ cell lines were plated in 6-well tissue culture plates previously coated with poly-2-hydroxyethyl-methacrylate (Sigma, St. Louis, MO) to prevent cell attachment, at a density of 1000 cells/mL in serum-free DMEM/F-12 supplemented with 1% L-glutamine, 1% penicillin/streptomycin, 2% B27 (Invitrogen, Carlsbad, CA), 20 ng/mL EGF (Sigma) and 20 ng/ mL FGFb (Invitrogen). The medium was made semi-solid by the addition of 0.5% methylcellulose (R&D Systems, Minneapolis, MN) to prevent cell aggregation. Mammosphere-forming efficiency (MSFE) was calculated as the number of sphere-like structures (diameter > 50 μm) formed after 7 days in the absence or presence of 1 mmol/L metformin, divided by the original number of cells seeded and expressed as a percentage (mean ± SD).

### Mitochondrial oxidation of glucose and glutamine

Oxidation rates of glucose and glutamine were measured using an XFp Extracellular Flux Seahorse Analyzer (Seahorse Bioscience). *BRCA1*^185delAG/+^ and *BRCA1*^+/+^ cells (15,000/well) were treated with 1 mmol/L metformin for 24 h prior to the star of the XFp Mito Fuel Flex Test kit, which was performed in accordance with manufacturer's instructions. Each plotted value is the mean of at least 6 replicates and is normalized to Hoechst signal in each well.

### PathScan sandwich immunoassay

The PathScan^®^ Intracellular Signaling array kit (Cell Signaling Technology, #7323) was used according to the manufacturer's instructions. Briefly, overnight serum-starved *BRCA1*^185delAG/+^ and *BRCA1*^+/+^ cells cultured in the absence or presence of 5 mmol/L metformin for 48 h in 5% HS were washed with ice-cold 1× phosphate-buffered saline and lysed in 1× Cell Lysis buffer. The Array Blocking Buffer was added to each well and incubated for 15 min at room temperature. Subsequently, the lysate was added to each well and incubated for 2 h at room temperature. Subsequent to washing, the detection antibody cocktail was added to each well and incubated for 1 h at room temperature. Horseradish peroxidase (HRP)-linked streptavidin was added to each well and incubated for 30 min at room temperature. The slide was then covered with ECL Clarity (Bio-Rad) and images were captured.

### Statistical analysis

Cell viability results are presented as the mean ± SD of at least three repeated individual experiments for each group. Two-group comparisons were performed using Student's *t* test for paired and unpaired values. In all cases, statistical analysis was carried out with XLSTAT (Addinsoft™) and *P* < 0.05 and *P* < 0.01 were considered significant. Results from targeted metabolomics were compared by one-way ANOVA with Dunnett's multiple pair-wise comparison tests using a significance threshold of 0.05. Other calculations including comparisons with the U of Mann-Whitney test and/or correlations were made using GraphPad Prism software 6.01 (GraphPad Software, San Diego, CA).
